# Disruption of Mitochondrial Bioenergetics Links Oxidative Stress-Mediated Apoptosis to Hepatorenal Injury Following Sub-Chronic Exposure to Titanium Dioxide Nanoparticles

**DOI:** 10.3390/ijms27198653

**Published:** 2026-09-28

**Authors:** Sara Bouzenzana, Rachid Rouabhi, Rosa Carotenuto, Chiara Fogliano, Simona Di Marino, Bice Avallone, Chiara Maria Motta

**Affiliations:** 1 Laboratory of Toxicology and Ecosystems Pathologies, Department of Living Beings, Echahid Cheikh Larbi Tebessi University, Tebessa 12002, Algeria; 2 Laboratory of Toxicology and Ecosystems Pathologies, Department of Applied Biology, Echahid Cheikh Larbi Tebessi University, Tebessa 12002, Algeria; r_rouabhi@univ-tebessa.dz; 3 Department of Biology, University of Naples Federico II, 80126 Naples, Italy; rosa.carotenuto@unina.it (R.C.); chiara.fogliano@unina.it (C.F.); simona.dimarino@unina.it (S.D.M.); bice.avallone@unina.it (B.A.); mottacm@unina.it (C.M.M.)

**Keywords:** mitochondrial bioenergetics, hepatorenal toxicity, caspase 3 activation, electron transport chain, nanotoxicology, titanium dioxide nanoparticles (TiO_2_-NPs)

## Abstract

Titanium dioxide nanoparticles (TiO_2_-NPs) are widely used nanomaterials, but their potential systemic toxicity following chronic exposure remains a significant concern. This study investigated whether mitochondrial dysfunction represents a key mechanistic link between oxidative stress, apoptosis, and organ injury induced by TiO_2_-NPs. Male Wistar rats were orally exposed for 90 days to TiO_2_-NPs (15–31 nm) at doses of 215 or 500 mg/kg body weight. Mitochondrial analyses revealed significantly impaired respiratory activity, increased membrane permeability, and mitochondrial swelling, indicating disruption of mitochondrial function and electron transport chain integrity. These alterations were accompanied by increased reactive oxygen species production; depletion of antioxidant defenses, including catalase (CAT), superoxide dismutase (SOD), glutathione peroxidase (GPx), reduced glutathione (GSH), and glutathione S-transferase (GST); and enhanced lipid peroxidation. Increased caspase-3 activation further suggested the involvement of mitochondria-dependent apoptotic pathways. Histopathological examination confirmed significant hepatorenal damage, including hepatocellular degeneration, inflammatory infiltration, vascular alterations, and glomerular and tubular lesions. Overall, integrating mitochondrial, oxidative, apoptotic, and histopathological findings supports a mechanistic model in which mitochondrial bioenergetic dysfunction acts as a central event linking oxidative imbalance to apoptosis and tissue injury. These findings contribute to a better understanding of TiO_2_-NP-associated health risks and provide mechanistic insights relevant to the safer development and application of nanomaterials.

## 1. Introduction

Nanotechnology and nanomaterials trace their conceptual origins back to 1959, when American theoretical physicist Richard Feynman presented the concept of atomic-scale matter manipulation in his groundbreaking talk “There’s Plenty of Room at the Bottom.” Although initially overlooked, this vision laid the foundation for modern nanotechnology, which gained significant scientific and industrial momentum in the 1980s [1]. Since then, nanotechnology has evolved into a transformative field with applications spanning medicine, electronics, energy, cosmetics, and the food industry.

Among engineered nanomaterials, titanium dioxide nanoparticles (TiO_2_-NPs) are the most widely produced, with a yearly market of about 23 billion dollars. Due to their physicochemical stability, photocatalytic activity, and relative biocompatibility, TiO_2_-NPs are employed in fields ranging from food production (food additive E171) to industrial processes [2]. Consequently, exposure to TiO_2_-NPs has become increasingly unavoidable, raising concerns about the potential adverse effects on biological systems [3].

Exposure in humans occurs mainly via ingestion of food products [4], but dermal [5] and respiratory routes are also consistent [6]. It has been calculated that the uptake of food-grade TiO_2_-NPs added to enhance color and brightness in food and toothpaste is about 4 μg/kg bw/day [7], while the estimated daily dermal exposure ranges from 2.8 to 21.4 mg TiO_2_ per person per day [8]. Further exposure derives from pharmaceuticals [9] and contaminated water. TiO_2_-NPs leak from food, paintings, coatings [10], and textiles [11]. Nanoparticles end up in the surface waters, where they reach concentrations up to several micrograms [12].

TiO_2_-NPs cross the gastrointestinal barrier and distribute to secondary organs, with the liver and kidneys as primary targets due to their central roles in metabolism, detoxification, and excretion. Nanoparticle accumulation in these organs causes structural and functional alterations and significant hepatorenal toxicity [13].

Oxidative stress is the central mechanism of TiO_2_-NPs-induced toxicity. Cellular redox equilibrium is disturbed by reactive oxygen species (ROS), and overwhelms antioxidant defense systems by unbalancing both enzymatic and non-enzymatic antioxidants, ultimately leading to lipid peroxidation, protein oxidation, and DNA damage [14,15].

Mitochondria play a pivotal role in TiO_2_ NP-induced toxicity, acting as a major source and a primary target of ROS. Mitochondrial dysfunction is characterized by depolarization of the mitochondrial membrane potential, impaired adenosine triphosphate (ATP) production, and altered protein expression [16]. In hepatocytes, mitochondrial membrane damage potential is possibly associated with increased rates of apoptosis [17]. Proapoptotic effects are supported by changes in mitochondrial proteins voltage-dependent anion-selective channel 1 (VDAC1) and sirtuin 3 (SIRT3), cytochrome c release, and subsequent caspase-3 activation [18].

At the tissue level, TiO_2_-NP-induced oxidative damage and mitochondrial dysfunction ultimately manifest as histopathological changes. These include cytoplasmic degeneration and nuclear damage, which disrupt tissue architecture in hepatocytes, kidneys, and brains, among others. These alterations, both directly and indirectly, promote necrosis and apoptosis, leading to progressive organ dysfunction.

Despite growing evidence linking TiO_2_-NPs to cellular and tissue damage, comprehensive in vivo studies that integrate these endpoints remain limited. Therefore, in this study, we aimed to investigate the effects of orally administered TiO_2_-NPs on the liver and kidneys of Wistar rats by assessing ROS generation, mitochondrial dysfunction, histopathological alterations, and apoptosis. This integrated approach seeks to provide deeper mechanistic insight into TiO_2_-NP-induced hepatorenal toxicity and to contribute to a more accurate risk assessment of the nanotoxicity of titanium dioxide nanoparticles after oral exposure.

## 2. Results

### 2.1. Oxidative Stress Markers in Liver and Kidney

Exposure to TiO_2_-NPs induced significant oxidative stress in both liver and kidney tissues, as shown in Table 1. In the liver, GST and GPx activities showed a highly significant decrease (215 mg/kg, *p* ≤ 0.01; 500 mg/kg, *p* ≤ 0.001) compared to controls, while CAT activity was also significantly reduced (215 mg/kg, *p* ≤ 0.01; 500 mg/kg, *p* ≤ 0.001). SOD activity showed a very highly significant decrease in the 215 mg/kg group (*p* ≤ 0.001) and a substantial reduction in the 500 mg/kg group (*p* ≤ 0.01). GSH levels were highly significantly decreased (215 mg/kg, *p* ≤ 0.01; 500 mg/kg, *p* ≤ 0.001), whereas malondialdehyde (MDA) levels showed a highly significant increase (215 mg/kg, *p* ≤ 0.01; 500 mg/kg, *p* ≤ 0.001), indicating lipid peroxidation and impaired antioxidant defenses.

Similarly, in the kidneys, GST and GPx activities were very highly significantly decreased in both treatment groups (215 mg/kg and 500 mg/kg, *p* ≤ 0.001), while CAT activity was highly significantly reduced (215 mg/kg and 500 mg/kg, *p* ≤ 0.01). SOD activity showed a very highly significant decrease in the 500 mg/kg group (*p* ≤ 0.001) and a highly significant decrease in the 215 mg/kg group (*p* ≤ 0.01). GSH levels were very highly significantly decreased (215 mg/kg and 500 mg/kg, *p* ≤ 0.001) and MDA levels were very highly significantly increased in both groups (215 mg/kg and 500 mg/kg, *p* ≤ 0.001), reflecting oxidative damage in renal tissue.

Overall, these results demonstrate that TiO_2_-NPs exposure disrupts the antioxidant defense system and promotes lipid peroxidation in both the liver and kidneys, supporting a mechanism of ROS-mediated organ toxicity.

### 2.2. Mitochondrial Function in Liver and Kidney

Exposure to TiO_2_-NPs markedly impaired mitochondrial function in both liver and kidney tissues.

In the liver (Figure 1A), mitochondrial respiration was significantly reduced, showing a very highly significant decrease in O_2_ consumption in the 500 mg/kg group (*p* ≤ 0.001) and a highly significant decrease in the 215 mg/kg group (*p* ≤ 0.01) compared to controls. Mitochondrial permeability increased by 136.67% in the 500 mg/kg group (*p* ≤ 0.001) and 95.56% in the 215 mg/kg group (*p* ≤ 0.001), while mitochondrial swelling was very highly significant in both treatment groups (*p* ≤ 0.001), indicating mitochondrial membrane destabilization as shown in Table 2.

In the kidneys (Table 2), mitochondrial swelling and permeability were highly significantly increased in both treatment groups. Mitochondrial permeability increased by 358.33% in the 500 mg/kg group (*p* ≤ 0.001) and by 291.67% in the 215 mg/kg group (*p* ≤ 0.001), while mitochondrial swelling was significantly elevated in both groups (*p* ≤ 0.01). Mitochondrial respiration (Figure 1B) showed a very highly significant decrease in O_2_ consumption in the 500 mg/kg group (*p* ≤ 0.001) and a significant reduction in the 215 mg/kg group (*p* ≤ 0.05) relative to controls. These findings demonstrate that TiO_2_-NPs exposure severely compromises mitochondrial integrity and bioenergetics in both organs, providing a mechanistic basis for downstream cellular damage and apoptosis (Table 2).

### 2.3. Effects of TiO_2_ Nanoparticles on Organ Anatomy

#### 2.3.1. Effects of TiO_2_ Nanoparticles on Kidney Anatomy

In the control group (Figure 2A,B), the kidney parenchyma displayed normal architecture, with intact glomeruli and proximal and distal tubules showing only occasional lumen swelling or minor epithelial alterations attributable to fixation artefacts. Following exposure to TiO_2_ nanoparticles, clear histological changes were observed, regardless of dose. The glomeruli appeared dense and congested (Figure 2C,D). Quantitative absorbance analysis indicated a 10.1% increase in glomerular density in animals treated with the lower dose (215 mg/kg). In contrast, the higher dose (500 mg/kg) showed a 6.6% decrease (Figure 2E), suggesting dose-dependent alterations in glomerular structure associated with nanoparticle exposure.

Congestion was also evident in the parenchyma (Figure 3). In control animals, blood vessels occupied only small spaces within the interstitial areas between tubules (Figure 3A,B). After TiO_2_-NPs exposure, vascular enlargement and congestion became more pronounced (Figure 3C,D). Morphometric analysis showed that the vessel area increased by 3.9% at 215 mg/kg and 5.2% at 500 mg/kg (Figure 3E), indicating a significant dose-related rise in vascular involvement after nanoparticle administration.

#### 2.3.2. Effects of TiO_2_ Nanoparticles on Liver Anatomy

In the control group, the liver exhibited a normal histoarchitecture characterized by well-organized hepatic lobules, without signs of fibrosis or sinusoidal congestion (Figure 4A). Hepatocytes displayed clear, homogeneous cytoplasm with small, dispersed lipid droplets. Their nuclei were centrally located, with dispersed chromatin and no indications of cellular degeneration (Figure 4B,C).

Following exposure to TiO_2_-NPs, notable histological alterations were observed. The most prominent change was the presence of large lipid droplets within hepatocytes exposed to 215 mg/kg (Figure 4D,E). At a higher concentration (500 mg/kg), marked sinusoidal dilation and cellular swelling were also evident (Figure 4F,G). Moreover, Kupffer cell numbers appeared to increase, suggesting activation of hepatic immune responses.

### 2.4. Effect of TiO_2_-NPs on Caspase 3 Expression

#### 2.4.1. Kidneys

Caspase-3 is poorly expressed in the control kidney (Figure 5), whereas the active fragment forms a dense band. After exposure to TiO_2_-NPs, caspase-3 expression increases significantly at the lower dose, as indicated by a 21.6% reduction in optical density (the 0 value corresponds to the maximum density; black in the grayscale). In contrast, at the higher dose, expression decreases, as indicated by a 16.9% increase in optical density (the 256 value corresponds to no density; white in the grayscale). Similarly, the active caspase-3 fragment is significantly reduced in both treated samples, with decreases of 27% and 11.4% at the lower and higher TiO_2_-NP doses, respectively.

#### 2.4.2. Liver

In the liver (Figure 6), the Western blot reveals that caspase 3 is poorly expressed in the control, while a notable amount of active caspase 3 is present. The absorbance values of the two bands have been determined, equated to 100% expression, and used to determine the relative expression in samples treated with TiO_2_-NPs.

At the lower dose of 215 mg/kg, caspase 3 expression decreased by 24.3%, while at the higher dose of 500 mg/kg, it increased by 16.5% compared with the control. The active caspase fragment did not vary significantly in the sample treated at the lower dose, while it increased by 31.1% in the sample exposed to the higher dose.

## 3. Discussion

Given the extensive applications of TiO_2_-NPs in industrial and biological settings, evaluating their potential health risks and underlying toxicological mechanisms is essential for proper risk assessment, safer use, and the development of nanotechnology-based applications. This study therefore examined the effects of subchronic exposure on mitochondrial function, apoptosis, and histopathological changes in the livers and kidneys of Wistar rats, aiming to elucidate the cellular and molecular mechanisms involved in TiO_2_-NP-induced toxicity under the experimental conditions used in this study. Although these findings provide evidence of associations among mitochondrial alterations, oxidative stress, apoptotic signaling, and tissue injury, they do not, by themselves, establish a direct causal sequence among these events.

In the present study, xanthine oxidase activity was increased in both the liver and kidneys following TiO_2_-NP exposure. This finding is consistent with previous evidence showing increased xanthine oxidoreductase activity following TiO_2_-NPs exposure [19], as well as with the reported prooxidant effects of TiO_2_-NPs [20,21] in both the liver [22] and the kidneys [23]. The significant, parallel increase in liver MDA indicates the induction of polyunsaturated fatty acid peroxidation [24], an alteration that would contribute to the reported DNA damage and protein peroxidation [25]. Taken together, these results suggest significant inflammation and cell damage [26]. However, because the present study did not directly quantify specific inflammatory mediators, these findings are more appropriately interpreted as consistent with oxidative and cellular injury.

Prooxidant responses in the liver differ from those in the kidney. Although xanthine levels in both tissues increase by about 10-fold, lipid peroxidation occurs in the liver, whereas in the kidneys, levels are even lower than in controls. These findings suggest that the response to this toxicant depends not only on exposure level but also on tissue-specific characteristics and susceptibility. However, the observed differences cannot be attributed solely to intrinsic organ-specific metabolic differences, particularly because quantitative measurements of TiO_2_-NP accumulation in the two tissues are missing. In the kidneys, lower GPx, CAT, and SOD activities indicate reduced antioxidant enzyme activity; however, the concomitant lower MDA level suggests that lipid peroxidation did not increase proportionally. This discrepancy indicates that additional tissue-specific factors may contribute to the observed redox response. These findings closely align with prior reports [27], which demonstrated that exposure to TiO_2_-NPs leads to pronounced oxidative stress and depletion of antioxidant defenses, especially in the liver. Rather, the present findings indicate that oxidative imbalance occurs alongside mitochondrial and apoptotic alterations, while previous studies provide evidence for possible mechanistic links among these processes [28].

It is known that NPs interact with all cell components. First, they interfere with cell membranes by altering cholesterol distribution [29] and membrane trafficking [30]. Relevantly, they attenuate macrophage phagocytic function, favoring NPs accumulation [31]. Membrane alterations may have particularly severe consequences for the kidney epithelial cells, which line the nephron and are designed for active transport, reabsorbing 99% of filtered water and essential nutrients back into the peritubular capillaries.

Once internalized, NPs are transported to lysosomes for biodegradation by acid hydrolases [32]. They may also remain in the cytoplasm or enter the nucleus, the mitochondria, and the endoplasmic reticulum. In the latter two cases, TiO_2_-NPs would generate ROS [33,34], further contributing to oxidative stress.

In mitochondria, TiO_2_-NPs significantly decrease ATP generation. The action is two-fold: by affecting cardiolipins, a class of phospholipids involved in the electron transport chain, and by interfering with the metabolic flux in the tricarboxylic acid cycle [35]. Consequently, blood glucose levels [33] and glycolysis [36] are altered. Mitochondrial impairment has already been observed in the brain of Wistar rats exposed to the same TiO_2_-NPs treatment used in the present study [37]. In this study, mitochondrial impairment in the liver and kidneys was evidenced by markedly reduced oxygen consumption, increased swelling, and enhanced membrane permeability, suggesting impaired mitochondrial respiratory function and increased electron leakage with ROS generation. This oxidative imbalance may contribute to oxidative damage to mitochondrial membrane lipids, respiratory proteins, and structural integrity, which may be associated with altered mitochondrial permeability and water influx into the mitochondrial matrix [38]. The swelling destabilizes the proton gradient and disrupts energy metabolism [39], leading to cell death via necrosis [40] or, through cytochrome c release, apoptosis [41]. Taken together, these findings support a possible ROS–mPTP pathway. However, because the present study did not directly measure ROS generation or experimentally inhibit mPTP opening, this pathway should be considered a plausible mechanism rather than a demonstrated causal relationship. Previous studies have nevertheless provided experimental evidence for ROS-dependent mPTP opening in TiO_2_-NPs-induced cytotoxicity [39].

Data collected on mitochondrial permeability indicates that the most severe effects of TiO_2_-NPs occur in the kidney, with values increasing 3-fold. This contrasts with the very low levels of GPx, SOD, and CAT, which instead suggest higher antioxidant defense, and with evidence that the kidneys, compared to the liver, accumulate fewer TiO_2_-NPs [42]. Differences in basal respiration rate [43] and/or metabolism (anaplerotic and cataplerotic pathways in the liver TCA cycle [44]) between the two tissues may contribute to this differential response. Although differences in organ physiology and metabolism may contribute to the observed responses, other factors, including tissue distribution, cellular uptake, antioxidant capacity, mitochondrial susceptibility, and clearance mechanisms, may also influence organ-specific toxicity. Further studies combining tissue titanium quantification with mechanistic analyses are therefore warranted to clarify the relationship between TiO_2_-NPs accumulation and organ-specific toxicity. The nephron is a highly specialized structure in which different segments exhibit distinct mitochondrial functions [45]. To fully understand the mitochondrial response, it is essential to determine mitochondrial dynamics. TiO_2_-NPs, in fact, can cause morphological changes and reduce fusion in the hepatocytes [46].

Given the mitochondrial dysfunction and ROS overproduction, activation of apoptotic pathways is an expected downstream outcome. Although no clear evidence was obtained at the cytological level [22,47], molecular investigations confirm a significant increase in active caspase-3 fragments, particularly at higher TiO_2_-NPs doses. Regarding procaspase-3, the kidneys and liver respond differently in terms of dose response. The kidney overexpresses caspase-3 at lower concentrations, whereas the liver shows overexpression only at higher doses. However, the kidneys showed altered caspase 3 expression, with reduced levels of cleaved fragments, potentially reflecting early mitochondrial injury, delayed apoptotic processing, or compensatory cytoprotective mechanisms that limit the complete execution of apoptosis. This phenomenon has been previously reported during prolonged nanoparticle exposure [48]. These data are difficult to reconcile with the observed levels of oxidative stress and antioxidant response. This supports the idea that mitochondrial damage is a central upstream regulator of the apoptotic pathway via sequential caspase activation [49]. Apoptosis magnitude is then modulated by cell susceptibility and/or TiO_2_ dose [50].

According to the literature, TiO_2_-NPs in the kidney also induce necrosis by activating nuclear factor-κB, thereby promoting the expression of tumor necrosis factor-α, macrophage migration inhibitory factor, and various inflammatory cytokines [51]. No evidence of necrosis was observed in our rats. This difference may reflect variations in dose, exposure duration, experimental conditions, or tissue susceptibility.

Oxidative stress and mitochondrial alterations appear to translate into histopathological changes in both the liver [26] and the kidneys. Congestion is the most evident effect in both the kidneys [52] and the liver [53]. In the latter, fatty degeneration was also observed [54], a damage concurring with lipid metabolism disorders [27] and glucose homeostasis imbalance [35]. Changes associated with altered lipid metabolism have been linked to interference with adipose tissue and to the onset of an inflammatory condition [55]. Currently, we lack sufficient quali–quantitative evidence of this interference, but the increase in Kupffer cells supports the hypothesis. Similarly, renal tissue exhibited dose-dependent structural disturbances, with lower doses showing increased glomerular density suggestive of an adaptive response to mild cellular stress, whereas higher doses produced marked degeneration, reduced glomerular density, dilation of Bowman’s capsule, and inflammatory infiltration [26]. In contrast, no fibrosis was observed, likely due to the relatively short exposure time [56]. These structural alterations are consistent with mitochondria-mediated oxidative injury progressing toward organ-level dysfunction, reinforcing the concept that mitochondrial damage represents an early initiating event driving oxidative imbalance, apoptotic signaling, inflammatory infiltration, and tissue pathology [26]. Taken together, the present findings support an association between mitochondrial dysfunction, oxidative stress, apoptotic signaling, and histopathological alterations following subchronic TiO_2_-NP exposure. Mitochondrial dysfunction may represent an important component of this response, but the available data do not establish it as the primary initiating event. The mechanistic links proposed here are therefore interpreted in light of evidence from previous studies. Further studies involving direct assessment of ROS generation, mPTP opening, mitochondrial dynamics, tissue TiO_2_ accumulation, and specific apoptotic signaling pathways are warranted to establish the causal relationships among these processes.

## 4. Materials and Methods

### 4.1. Experimental Design

In this study, thirty rats, aged 4–6 months and weighing 220–260 g, were obtained from the Algerian Pasteur Institute (API), Algiers, Algeria. They were randomly assigned to three groups of 10 animals each (*n* = 10 per group). The study was approved by the local Consultative Ethics Committee (CCE) of the Biotechnology Research Centre (CRBT), Constantine, Algeria. Under the Algerian Ministry of Higher Education and Scientific Research (protocol code N09HA-2021/2023/CCE, Date of approval: 9 September 2021), and was conducted in accordance with the ARRIVE and OECD guidelines [57].

The TiO_2_-NPs used in the present study were sourced from the University of El-Oued, El-Oued city, Algeria. Their physicochemical properties are detailed in our previous study investigating their neurotoxic effects [37]. In particular, morphology, primary particle size, crystallinity, crystalline phase composition, and elemental composition are reported. The nanoparticles were identified as phase-pure anatase TiO_2_, with an average primary particle size of approximately 31 nm and a width distribution (SD = 15 nm). TiO_2_-NPs were dissolved in 1 mL of distilled water at pH 7.4 (expected zeta potential ranging from −10 to −20 mV). Administration was via oral gavage and lasted for 90 consecutive days. Group 1 (control) received 1 mL of distilled water, while the two treatment groups received TiO_2_-NPs at 215 or 500 mg/kg body weight/day, dissolved in 1 mL of distilled water, respectively.

Rats were housed under standard laboratory conditions with free access to food and water. The detailed exposure protocol and nanoparticle preparation are described in our earlier work [37].

### 4.2. Dose Selection

The doses used in the present study were selected based on median lethal dose (LD_50_) data reported in the Fiche Toxic and by the Scientific Committee on Consumer Safety [58], including data from previous studies [59,60]. The selected doses were adjusted to ensure animal survival throughout the experimental period while maintaining toxicological relevance and scientific validity. These doses were selected to investigate sub-chronic TiO_2_-NP toxicity and to better characterize the underlying biological and molecular mechanisms, particularly the interplay between oxidative stress, mitochondrial dysfunction, and apoptosis. Importantly, the selected doses were not intended to represent realistic human exposure levels but rather to investigate toxicological responses and mechanisms under controlled experimental conditions.

### 4.3. Tissue Preparations

After 90 days of exposure, rats were anaesthetized by brief inhalation of chloroform vapor generated from a small volume of liquid chloroform applied to cotton within a closed container. Exposure was limited to a few seconds until loss of the righting reflex. While deeply anaesthetized, the animals were euthanized by terminal blood collection via cardiac puncture. Procedures were approved by the local Ethics Committee of the Biotechnology Research Centre (CRBT), under the Algerian Ministry of Higher Education and Scientific Research, and were conducted in accordance with the ARRIVE and OECD guidelines. Upon confirmation of death, liver and kidney samples were dissected and either immediately used for mitochondrial extraction or processed for light microscopy. Portions intended for oxidative stress analyses were stored at −20 °C until use. For homogenization, 200 mg of liver or kidney tissue was suspended in 2 mL of 100 mM phosphate buffer (pH 7.5) and homogenized using a Vibra Cell VCX 750 sonicator (Sonics & Materials Inc., Newtown, CT, USA). Sonication was performed in 15 s on/10 s off cycles for a total duration of 60–90 s at 600 [unit]. The homogenates were then centrifuged at 3000× *g* for 15 min at 4 °C, and the resulting supernatants were collected for subsequent biochemical and molecular analyses.

### 4.4. Mitochondrial Isolation

The organs were homogenized in an isolation buffer (210 mM Mannitol, 70 mM sucrose, 1 mM Tris-HCl, 1 mM EDTA, pH 7.5) using a Potter–Elvehjem homogenizer (Bellco Glass Inc., Vineland, NJ, USA). The homogenate was centrifuged at 4 °C, 1300× *g* for 3 min, and the recovered supernatant was centrifuged again at 10,000× *g* for 10 min to pellet mitochondria. The pellet was washed twice in the isolation buffer and then suspended in a second buffer (0.23 M Mannitol, 0.07 M Sucrose, 20 mM Tris-HCl, pH 7.4, 1 mM EDTA, 5 mM phosphate, 4 mM MgCl_2_). Protein content was routinely assayed by Bradford assay using bovine serum albumin as a standard [61]. Mitochondria were kept on ice and used within 4 h.

### 4.5. Evaluation of Mitochondrial Respiration, Permeability, and Swelling

Oxygen consumption was evaluated as described in [62], using a Clark-type oxygen electrode system (HANSATECH Instruments Ltd., Norfolk, UK) coupled with an Oxygraph+ control unit. Briefly, 1 mL of respiration buffer was used to suspend 1 milligram of isolated mitochondria (0.23 M Mannitol, 0.07 M Sucrose, 20 mM Tris–HCl (pH 7.4), 1 mM EDTA, 5 mM Phosphate, and 4 mM MgCl_2_) and transferred into the incubation chamber. Oxygen consumption was continuously monitored for 20 min, with ADP (200 µL, 1 mM) and Succinate (200 µL, 10 mM) added at 4 and 10 min, respectively.

Membrane permeability and mitochondrial swelling were assessed according to [63], using Ca^2+^ to induce the mitochondrial permeability transition (mPTP). Swelling was measured spectrophotometrically at 540 nm every 30 s for 2 min. The swelling rate was estimated from the slope of the absorbance decrease, which served as a marker of mitochondrial membrane integrity.

### 4.6. Assessment of Oxidative Stress Markers in Liver and Kidneys

The redox status in hepatic and renal tissues was evaluated by lipid peroxidation mensuration and antioxidant enzyme activities. Lipid peroxidation was assessed by measuring malondialdehyde (MDA) levels were determined based on the reaction of MDA with thiobarbituric acid (TBA). Briefly, 375 µL of tissue supernatant was mixed with 375 µL of TCA-BHT solution (20% TCA, 1% BHT), then centrifuged at 1000× *g* for 10 min. Then, 400 µL of the resulting supernatant was mixed with 80 µL of HCl (0.6 M) and 320 µL of Tris-TBA solution (26 mM Tris, 120 mM TBA), incubated at 80 °C for 10 min, and the absorbance was measured at 530 nm against an appropriate blank [64].

The enzymatic antioxidant defense system was evaluated by measuring SOD activity was measured using the method described by [65]. Using the pyrogallol autoxidation method. The reaction mixture contained 880 µL of Tris/DTPA buffer (50 mM, pH 8.2, containing 1 mM Diethylenetriaminepentaacetic acid DTPA), 80 µL of tissue supernatant, and 40 µL of pyrogallol (10 mM). Following a 45 s reaction period, absorbance was monitored at 420 nm for 1 min. The assay was performed in duplicate, and SOD activity was expressed as units/mg protein.

CAT activity was assessed by monitoring H_2_O_2_ decomposition. Briefly, 100 µL of tissue supernatant was mixed with 50 µL of H_2_O_2_ and 2850 µL of phosphate buffer (50 mM, pH 7.2). Absorbance was measured at 240 nm after a 15 s equilibration period, then every 5 s for 30 s. CAT activity was expressed as nmol/min/mg protein [66].

GST activity was determined according to the method of [67] using the CDNB/GSH conjugation reaction. Briefly, 200 µL of tissue supernatant was added to 1.2 mL of CDNB/GSH solution. Absorbance was measured at 340 nm every minute for 5 min against an appropriate blank. The results were expressed as pmol/min/mg protein.

GPx activity was determined based on [68] using GSH and H_2_O_2_ as substrates. Briefly, 200 µL of tissue supernatant was mixed with 400 µL of GSH (0.1 mM) and 200 µL of phosphate buffer (0.067 M, pH 7.8), then incubated at 25 °C for 5 min. The reaction was initiated by adding 200 µL of H_2_O_2_ (1.3 mM) and allowed to proceed for 10 min, after which 1 mL of TCA (1%) was added to stop the reaction. After incubation on ice for 30 min and centrifugation at 3000× *g* for 10 min, 480 µL of supernatant was mixed with 2.2 mL of Na_2_HPO_4_ (0.32 M) and 320 µL of DTNB (1 mM). Absorbance was measured at 412 nm every 30 s for 5 min. The results were expressed as nmol/min/mg protein.

GSH content was determined as described in [69]. Levels were determined by measuring the reaction of reduced glutathione with DTNB. Briefly, 0.8 mL of tissue homogenate was mixed with 0.2 mL of sulfosalicylic acid (ASS) and kept on ice for 15 min, then centrifuged at 1000× *g* for 5 min. Subsequently, 500 µL of the supernatant was mixed with 1 mL of Tris/EDTA buffer (0.02 M, pH 9.6), followed by the addition of 25 µL of DTNB (0.01 M). After 5 min for color stabilization, absorbance was measured at 412 nm against a reagent blank. The results were expressed as nmol/mg of protein.

All enzyme activities were expressed relative to the protein content of the corresponding tissue samples.

### 4.7. Histological Investigations

The tissue samples (*n* = 3 per treatment) were fixed in 4% paraformaldehyde, dehydrated through a graded series of ethanol, and cleared with methyl benzoate (3 days) to remove residual alcohol and aqueous traces. The tissues were then washed in xylene and embedded in paraffin wax (4 h, with two changes). Paraffin blocks were sectioned into 6 µm-thick slices using a microtome. The two organs were processed across their available tissue extent, and all sections obtained (about 500 per sample) were examined to assess tissue architecture and pathological alterations.

For staining, sections were deparaffinized in xylene and rehydrated through descending ethanol alcohol concentrations. Hematoxylin was applied for 15 min to visualize nuclei, followed by 0.25% eosin for 20 s to stain the cytoplasmic components and provide optimal contrast for cellular and tissue morphology [70]. The stained sections were examined under a Zeiss Axioskop microscope (Carl Zeiss, Jena, Germany), and digital micrographs were captured using a Zeiss Axiocam imaging system (Carl Zeiss, Jena, Germany).

Semithin sections were prepared from tissues (*n* = 3 samples per treatment) fixed in 4% paraformaldehyde and post-fixed in osmium tetroxide for 1 h. After dehydration in graded ethanol at 4 °C, tissues were clarified in propylene oxide and embedded in Epon 812 [71]. Semithin sections (>75/sample), 1 µm thick, were stained with toluidine blue [70].

### 4.8. Western Blot Analysis of Caspase-3 Expression

Western blot analyses were performed on total liver and kidney homogenates to assess caspase-3 expression. For protein extraction, tissues were homogenized in HB buffer containing 900 mM glycerol, 0.02 mM NaN_3_, 1 mM ATP, 1 mM DTT, and 5 mM EDTA, supplemented with a protease inhibitor cocktail (Sigma-Aldrich, St. Louis, MO, USA) containing 2 mM TAME, 5 mg/mL soybean trypsin inhibitor (SBTI), 5 mg/mL aprotinin, and 10 mM PMSF. Homogenates were centrifuged at 13,000× *g* for 40 min at 4 °C, and the supernatants were collected for protein analysis. Protein concentration in the supernatants was determined using the BCA Protein Assay Reagent (Pierce, Rockford, IL, USA) according to the manufacturer’s instructions.

Prior to electrophoresis, samples were boiled in sample buffer containing 50 mM Tris-HCl (pH 6.8), 69 mM SDS, 0.028 mM bromophenol blue, and 1 mM DTT. A total of 60 µg of protein from each sample was separated by 10% SDS-polyacrylamide gel electrophoresis (SDS-PAGE) using protein molecular weight standards as reference markers (200, 116, 97, 45, 31, and 21 kDa; Bio-Rad, Hercules, CA, USA). Proteins were then electrotransferred onto nitrocellulose membranes overnight at 200 mA in transfer buffer containing 25.1 mM Tris base, 192 mM glycine, and 13.5% (*v*/*v*) methanol, as previously described in [72].

Following transfer, membranes were washed three times for 5 min each with TBS (9.9 mM Tris and 154 mM NaCl) and stained with Ponceau S solution (14,408,162, Longlac-Rozzano, Italy) twice for 3 min to verify protein transfer. Membranes were subsequently washed and blocked overnight at 4 °C in 3% BSA (Sigma, A2153-50G) prepared in TBS.

For caspase 3 detection, membranes were incubated with a rabbit polyclonal anti-caspase-3 active antibody (Sigma-Aldrich, C8487; 1:500). Following washing, membranes were incubated with an alkaline phosphatase-conjugated anti-rabbit IgG secondary antibody (Sigma-Aldrich, A3687; 1:20,000).

Immunoreactive bands were visualized with a BCIP/NBT chromogenic substrate system (Sigma-Aldrich). The developed membranes were imaged with a ChemiDoc imaging system (Bio-Rad, Milan, Italy).

### 4.9. Data Analysis

All experimental data were analyzed using IBM SPSS Statistics version 20. Results were assessed for normality using the Shapiro–Wilk test and reported as mean ± standard deviation (SD). Differences between groups were evaluated using one-way ANOVA with Tukey’s post hoc test (GraphPad Prism 5.0, GraphPad Software, CA, USA). A *p*-value < 0.05 was considered significant.

Histological parameters and blot band densities were quantified using ImageJ (version 1.54r, 25 September 2025; https://imagej.net/ij/download.html) (accessed on 15 October 2025). The greyscale ranged from 0 (black, higher density) to 256 (white, no density). For glomerular and vascular density analyses, random digital micrographs were acquired at 20× magnification and then converted to high-resolution (400 dpi), 8-bit grayscale TIFF images. Mean absorbance was measured in *n* = 50 randomly selected areas per treatment group (see Figure 2D and Figure 3D) using the Analyze > Measure option [73]. Blots were acquired directly as high-resolution (400 dpi) TIFF images. Band densities were determined using the Analyze > Measure option. Measurements were normalized by calculating the Lane Normalization Factor (LNF). In brief, lane band densities were corrected by a factor derived from the total protein loaded per lane. Graphs and histograms were generated in OriginLab 2025.

## 5. Conclusions

In conclusion, the current findings provide a unified mechanistic model in which mitochondrial dysfunction caused by TiO_2_-NPs acts as the initial event linking oxidative stress, metabolic imbalance, caspase-dependent apoptosis, and histopathological damage in the liver and kidneys. The extent of the damage is considerable, even with subchronic exposure. This suggests that greater attention should be paid to the release of TiO_2_-NPs into the environment, especially in contexts where their use may lead to dietary intake.

## Figures and Tables

**Figure 1:**
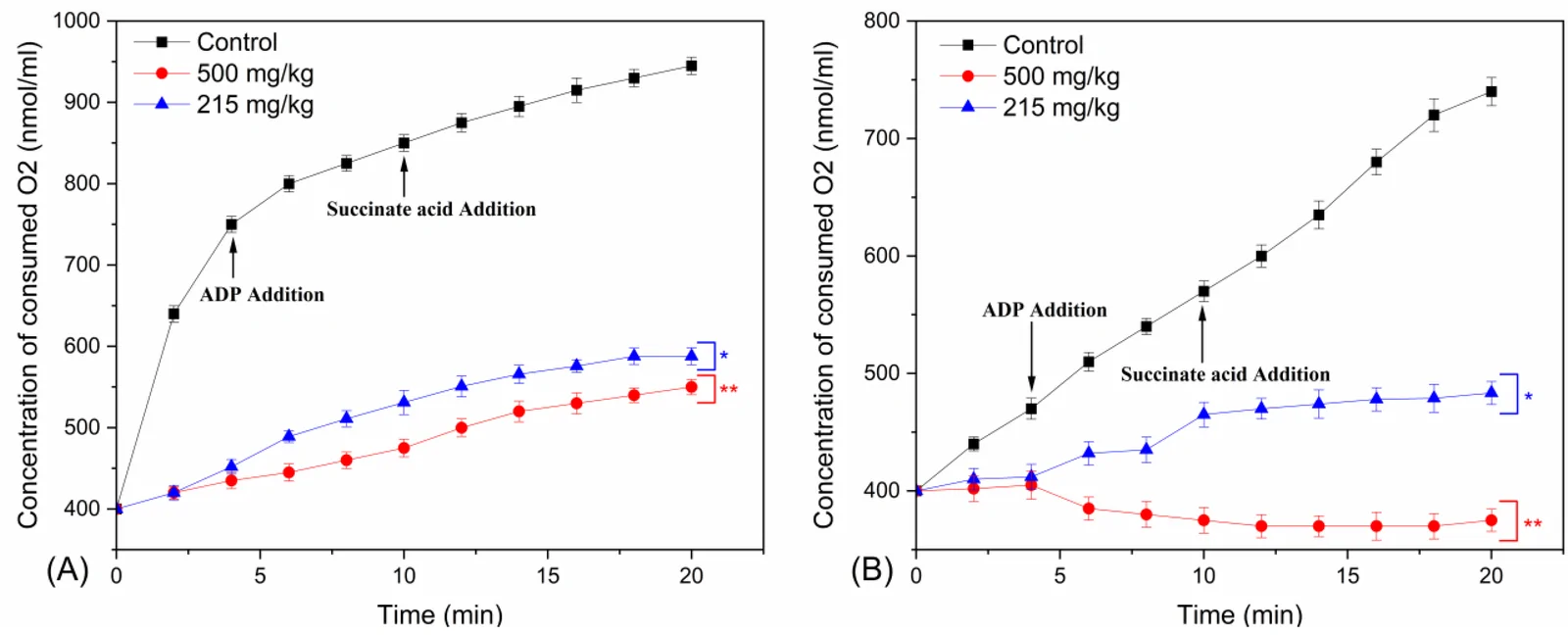
Effect of oral administration of TiO_2_ nanoparticles on mitochondrial respiration in the liver (**A**) and kidney (**B**). Values are expressed as means ± standard deviations, *n* = 10 rats per group. ANOVA followed by Tukey’s post hoc test. Statistical significance is indicated as follows: significant (*, *p* ≤ 0.05); highly significant (**, *p* ≤ 0.01).

**Figure 2:**
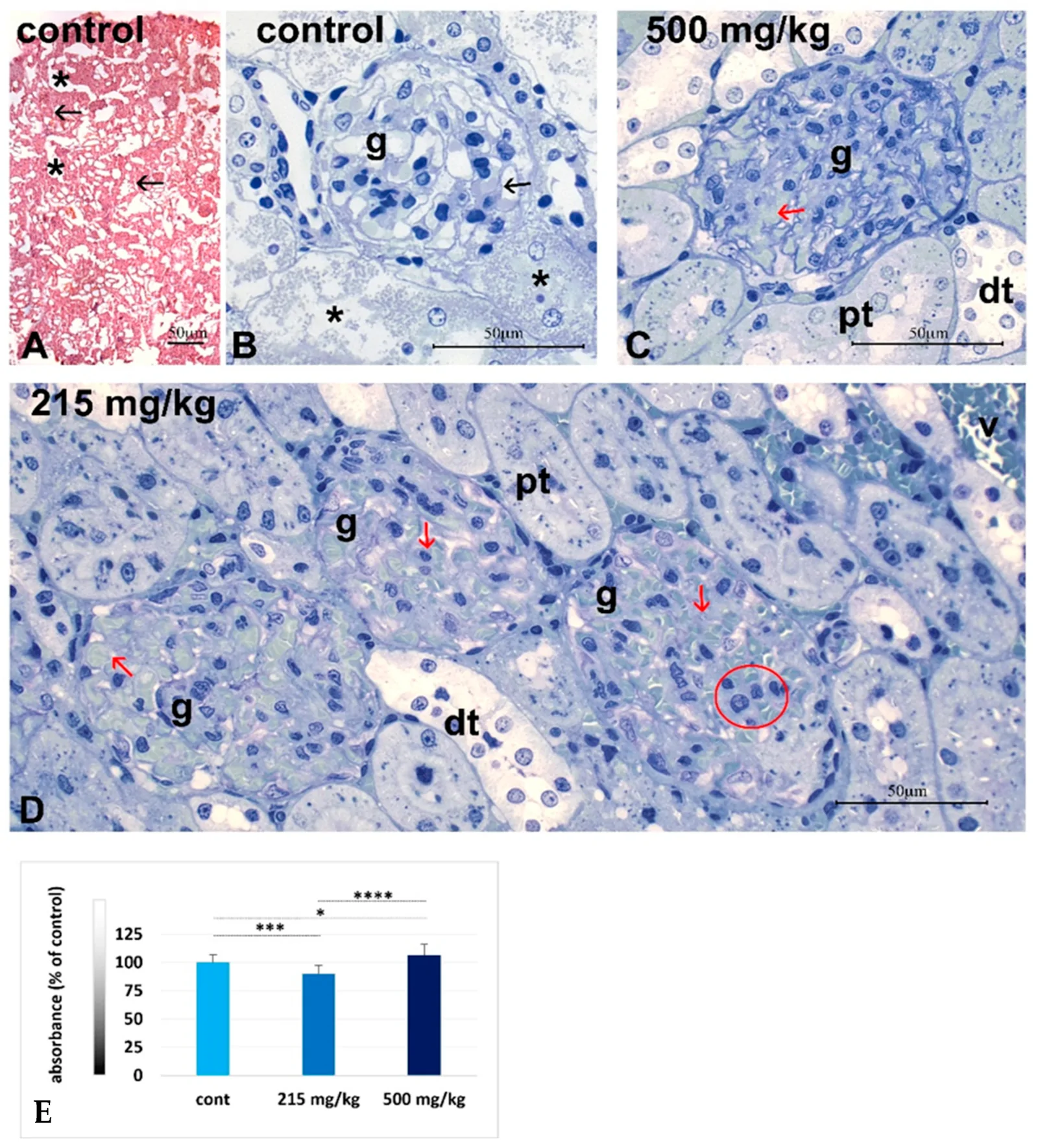
Effects of TiO_2_ nanoparticles on kidney parenchyma. (**A**,**B**) General aspect. Notice the moderately swollen tubules (*) and the presence of a few artefacts (arrows). (**B**) Detail of an intact glomerulus (g). Blood cells (arrow) and moderately altered tubules (*). (**C**) Congested glomerulus filled with blood cells (arrow). (**D**) Parenchyma with intact proximal (pt) and distal (dt) tubules and congested glomeruli (g). Haemalum-eosin staining (**A**); semi-thin sections stained with toluidine blue (**B**–**D**). (Number of animals examined, *n* = 3/treatment). (**E**) Optical density of glomeruli, measured in areas as indicated by the red circle in (**D**). Values are expressed as a percentage of the control value. Greyscale: 0 = black, dense glomerulus; 256 = white, clear glomerulus. Two-way ANOVA followed by Tukey’s pairwise comparison test. Statistical significance is indicated as follows: significant (*, *p* < 0.05); highly significant (***, *p* < 0.001); very highly significant (****, *p* < 0.0001). (*n* = 50 glomeruli/treatment).

**Figure 3:**
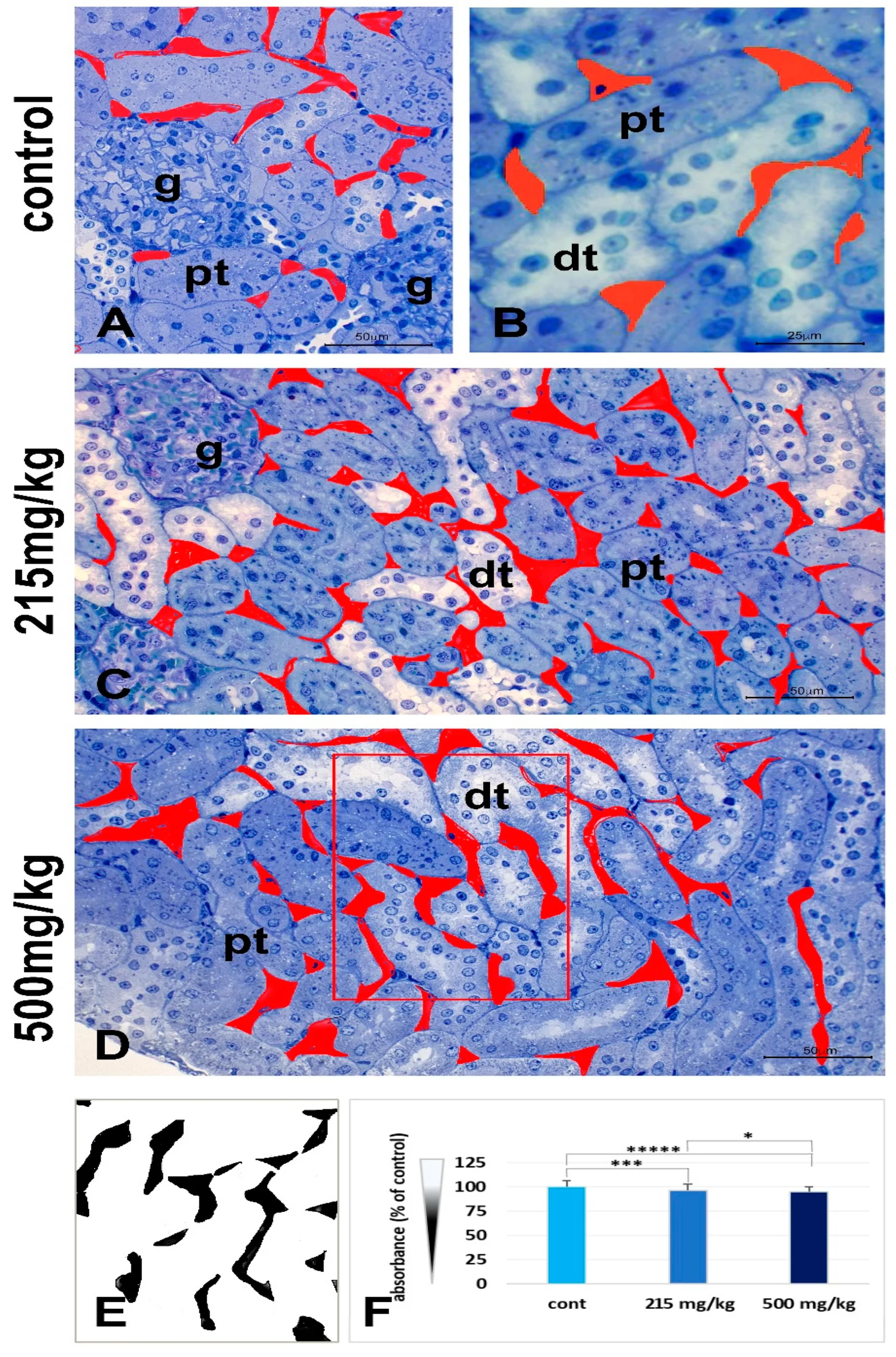
Vascular congestion in the renal parenchyma following oral exposure to TiO_2_ nanoparticles. Representative histological sections of kidney parenchyma showing blood vessel distribution and congestion. (**A**,**B**) Control group. Vessels occupy only small interstitial spaces between renal tubules. (**C**) Treated group, 215 mg/kg. Vascular congestion. (**D**) Treated group, 500 mg/kg. Pronounced congestion. The red box represents the areas examined to determine absorbance. (**E**) Quantitative morphometric analysis revealed increases of 3.9% at 215 mg/kg and 5.2% at 500 mg/kg in the area occupied by blood vessels compared with controls. (**F**) Optical density measured in areas as indicated by the red box in (**D**). Greyscale: 0 = black, dense glomerulus; 256 = white, clear glomerulus. Two-way ANOVA followed by Tukey’s pairwise comparison test. Statistical significance is indicated as follows: significant (*, *p* < 0.05); highly significant (***, *p* < 0.001); extremely significant (*****, *p* < 0.00001). (*n* = 50 areas/treatment).

**Figure 4:**
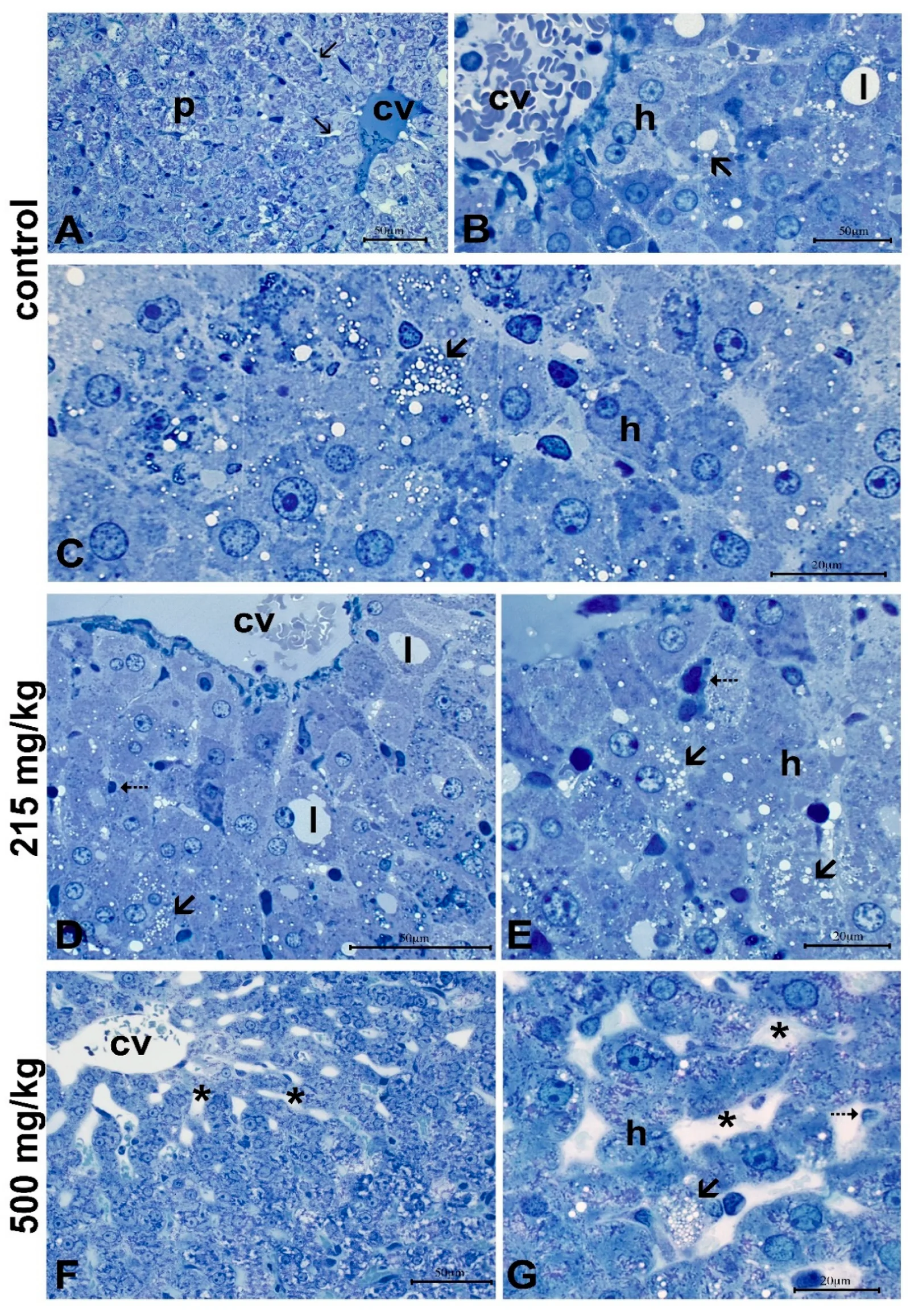
Effects of TiO_2_ nanoparticles on liver parenchyma. (**A**) Parenchyma (p) with a centrilobular vein (cv). Sinusoids (thin arrows). (**B**,**C**) Details showing the presence of small (arrows) and occasional large (l) lipid droplets in hepatocytes (h). (**D**,**E**) Presence of small (arrows) and occasional large (l) lipid droplets in hepatocytes (h). Kupffer cells (dotted arrows). (**F**,**G**) Enlarged sinusoids (*) and presence of small lipid droplets (arrow) in hepatocytes (h). Semithin sections, stained with toluidine blue. (Number of animals examined, *n* = 3/treatment).

**Figure 5:**
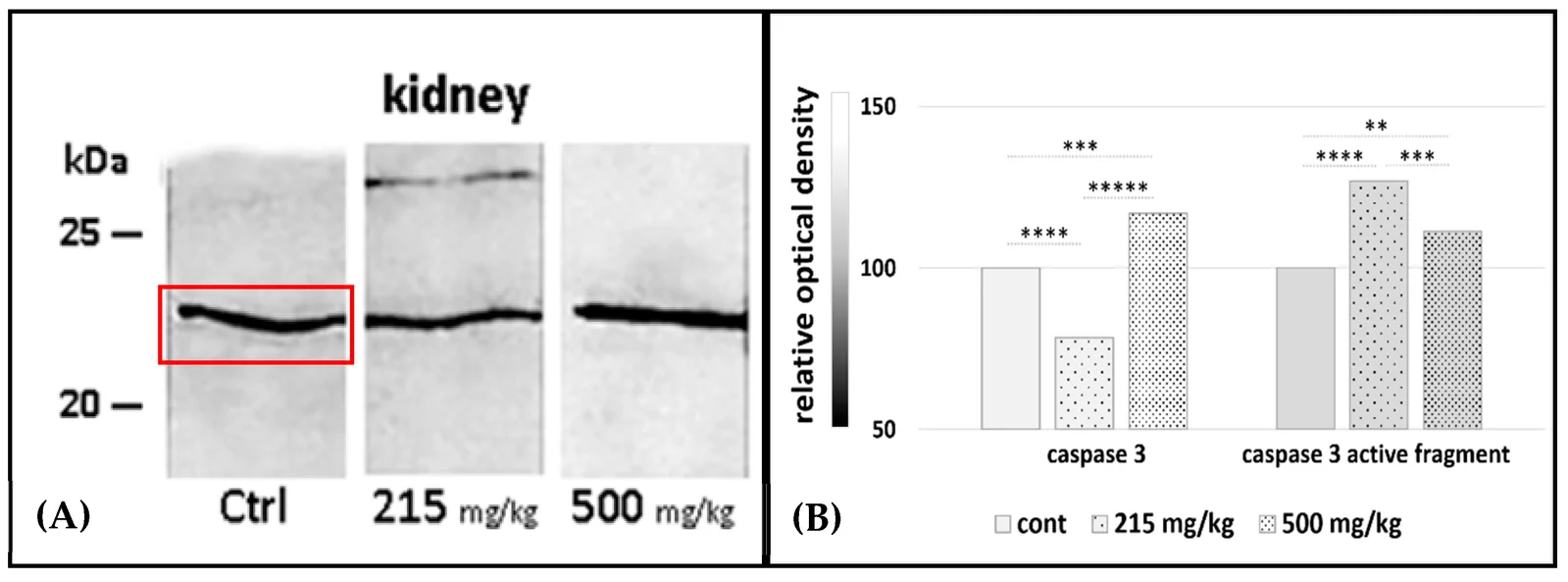
Changes in caspase 3 expression in the kidneys exposed to TiO_2_ nanoparticles. (**A**) Western blotting; upper band: caspase 3; lower band: cleaved fragment of caspase 3. Number of animals examined, *n* = 3; number of blots examined, *n* = 1. (**B**) Optical density (grey values) of bands shown in (**A**). The red square indicates the measured area. Greyscale: 0 = black, dense band; 256 = white, less dense band. Statistical significance is indicated as follows: very significant (**, *p* < 0.01); highly significant (***, *p* < 0.001); very highly significant (****, *p* < 0.0001); extremely significant (*****, *p* < 0.00001).

**Figure 6:**
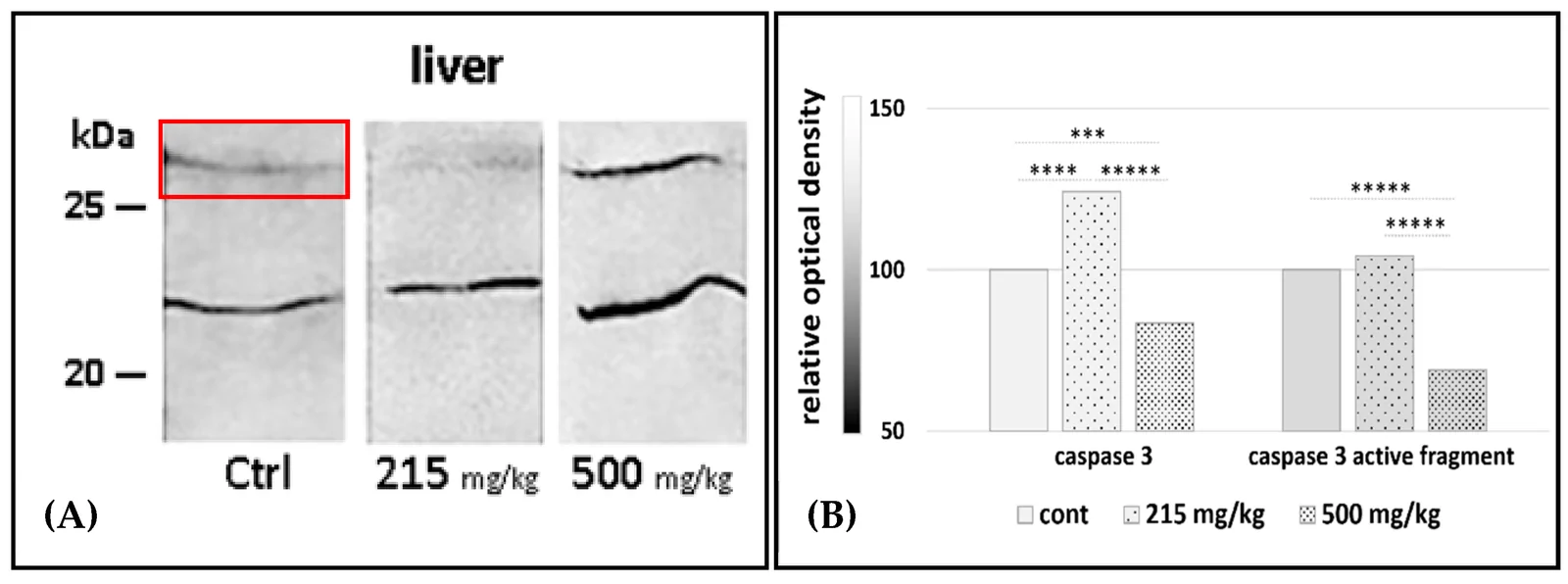
Changes in caspase 3 expression in the liver exposed to TiO_2_ nanoparticles. (**A**) Western blotting; upper band: caspase 3; lower band: cleaved fragment of caspase 3. Number of animals examined: *n* = 3; number of blots: *n* = 1. (**B**) Optical density (grey values) of bands shown in (**A**). The red square indicates the measured area. Greyscale: 0 = black, dense band; 256 = white, less dense band. Statistical significance is indicated as follows: highly significant (***, *p* < 0.001); very highly significant (****, *p* < 0.0001); extremely significant (*****, *p* < 0.00001).

**Table 1:** Effect of oral administration of TiO_2_-NPs on redox markers in the liver and kidneys.

		Liver		Kidneys	
Redox Status Markers	Control	TiO_2_-NPs 500 mg/kg/day	TiO_2_-NPs 215 mg/kg/day	TiO_2_-NPs 500 mg/kg/day	TiO_2_-NPs 215 mg/kg/day
GSH nmol/mg	9.01 ± 0.01	3.11 ± 0.01 ***	4.53 ± 0.01 ***	5.68 ± 1.45 ***	9.77 ± 2.08 ***
GST pmol/min/mg	1.79 ± 0.08	0.30 ± 0.02 ***	0.43 ± 0.03 ***	0.43 ± 0.09 ***	0.49 ± 0.08 ***
SOD U/mg	2.85 ± 0.11	2.35 ± 0.33 **	1.72 ± 0.17 ***	1.50 ± 0.41 ***	1.86 ± 0.60 ***
CAT nmol/min/mg	12.57 ± 0.01	3.99 ± 0.01 ***	4.67 ± 0.01 ***	0.01 ± 0.00 **	0.01 ± 0.00 **
MDA nmol/mg	29.77 ± 2.05	37.03 ± 6.30 *	35.85 ± 4.76 *	21.96 ± 6.35 ***	17.17 ± 3.54 ***
GPX nmol/min/mg	8.56 ± 0.82	3.64 ± 1.12 ***	2.68 ± 0.88 ***	0.10 ± 0.06 ***	0.14 ± 0.03 ***
Xanthine (XO)	0.02 ± 0.00	0.24 ± 0.00 ***	0.19 ± 0.01 ***	0.20 ± 0.09 ***	0.15 ± 0.02 ***

Each value is expressed as mean ± standard deviation; *n* = 10 rats per group. ANOVA followed by Tukey’s post hoc test. Statistical significance is as follows: significant (*, *p* ≤ 0.05); highly significant (**, *p* ≤ 0.01); very highly significant (***, *p* ≤ 0.001).

**Table 2:** Effect of oral administration of TiO_2_-NPs on mitochondrial swelling and permeability in the liver, and kidney tissues.

		Liver		Kidney	
Variable	Control Group	TiO_2_-NPs 500 mg/kg/day	TiO_2_-NPs 215 mg/kg/day	TiO_2_-NPs 500 mg/kg/day	TiO_2_-NPs 215 mg/kg/day
Swelling rate ΔA 540/min	0.09 ± 0.03	0.213 ± 0.08 ***	0.176 ± 0.1 ***	0.275 ± 0.01 ***	0.235 ± 0.03 ***
Permeability (%)		136.67% ***	95.56% ***	358.33% ***	291.67% ***

Values are means ± standard deviations, *n* = 10 rats per group. ANOVA followed by Tukey’s post hoc test. Statistical significance is indicated as follows: very highly significant (***, *p* ≤ 0.001).

## Data Availability

The data presented in this study are available from the corresponding author upon reasonable request.

## Abbreviations

The following abbreviations are used in this manuscript:

TiO_2_-NPs	Titanium Dioxide Nanoparticles
GST	Glutathione S-transferase
GSH	Reduced glutathione
GPx	Glutathione peroxidase
SOD	Superoxide dismutase
CAT	Catalase
MDA	Malondialdehyde
